# Categorizing Health Outcomes and Efficacy of mHealth Apps for Persons With Cognitive Impairment: A Systematic Review

**DOI:** 10.2196/jmir.7814

**Published:** 2017-08-30

**Authors:** Daniel R Bateman, Bhavana Srinivas, Thomas W Emmett, Titus K Schleyer, Richard J Holden, Hugh C Hendrie, Christopher M Callahan

**Affiliations:** ^1^ Indiana University Center for Aging Research Regenstrief Institute, Inc Indianapolis, IN United States; ^2^ Department of Psychiatry Indiana University School of Medicine Indianapolis, IN United States; ^3^ School of Informatics and Computing Indiana University-Purdue University Indianapolis Indianapolis, IN United States; ^4^ Ruth Lilly Medical Library Indiana University School of Medicine Indianapolis, IN United States; ^5^ Department of Medicine Indiana University School of Medicine Indianapolis, IN United States; ^6^ Center for Biomedical Informatics Regenstrief Institute, Inc Indianapolis, IN United States

**Keywords:** mHealth, mobile health, applications, Alzheimer disease, dementia, systematic review

## Abstract

**Background:**

Use of mobile health (mHealth) apps is growing at an exponential rate in the United States and around the world. Mild cognitive impairment (MCI), Alzheimer disease, and related dementias are a global health problem. Numerous mHealth interventions exist for this population, yet the effect of these interventions on health has not been systematically described.

**Objective:**

The aim of this study is to catalog the types of health outcomes used to measure effectiveness of mHealth interventions and assess which mHealth interventions have been shown to improve the health of persons with MCI, Alzheimer disease, and dementia.

**Methods:**

We searched 13 databases, including Ovid MEDLINE, PubMed, EMBASE, the full Cochrane Library, CINAHL, PsycINFO, Ei Compendex, IEEE Xplore, Applied Science & Technology Source, Scopus, Web of Science, ClinicalTrials.gov, and Google Scholar from inception through May 2017 for mHealth studies involving persons with cognitive impairment that were evaluated using at least one quantitative health outcome. Proceedings of the Annual ACM Conferences on Human Factors in Computing Systems, the ACM User Interface Software and Technology Symposium, and the IEEE International Symposium on Wearable Computers were searched in the ACM Digital Library from 2012 to 2016. A hand search of JMIR Publications journals was also completed in July 2017.

**Results:**

After removal of duplicates, our initial search returned 3955 records. Of these articles, 24 met final inclusion criteria as studies involving mHealth interventions that measured at least one quantitative health outcome for persons with MCI, Alzheimer disease, and dementia. Common quantitative health outcomes included cognition, function, mood, and quality of life. We found that 21.2% (101/476) of the fully reviewed articles were excluded because of a lack of health outcomes. The health outcomes selected were observed to be inconsistent between studies. For those studies with quantitative health outcomes, more than half (58%) reported postintervention improvements in outcomes.

**Conclusions:**

Results showed that many mHealth app interventions targeting those with cognitive impairment lack quantitative health outcomes as a part of their evaluation process and that there is a lack of consensus as to which outcomes to use. The majority of mHealth app interventions that incorporated health outcomes into their evaluation noted improvements in the health of persons with MCI, Alzheimer disease, and dementia. However, these studies were of low quality, leading to a grade C level of evidence. Clarification of the benefits of mHealth interventions for people with cognitive impairment requires more randomized controlled trials, larger numbers of participants, and trial designs that minimize bias.

**Trial Registration:**

PROSPERO Registration: PROSPERO 2016:CRD42016033846; http://www.crd.york.ac.uk/PROSPERO/ display_record.asp?ID=CRD42016033846 (Archived by WebCite at http://www.webcitation.org/6sjjwnv1M)

## Introduction

Industry analysts expect worldwide mobile phone app users to increase from 2.6 billion in 2015 to 6.1 billion users by 2020 [1,2]. Similarly, analysts forecast worldwide mobile device app downloads and usage to grow from 111.2 billion in 2015 to 284.3 billion by 2020 [3]. From a financial scope, global mobile app gross revenue for 2016 surpassed US $51 billion and by 2020 is expected to exceed US $101 billion [3]. The National Institutes of Health (NIH) Consensus Group on mHealth defines mobile health (mHealth) as “the use of mobile and wireless devices to improve health outcomes, health care services, and health research” [4,5]. The NIH Strategic Plan for 2016-2020 incorporates the study of mHealth technologies and their ability to help prevent and treat illness as a research priority [6]. Following the aforementioned NIH definition of mHealth, a mHealth app operates on either a mobile or wireless device, with an objective of improving health outcomes, health care services, or health research [4].

The mHealth technologies and apps that help persons with mild cognitive impairment (MCI), Alzheimer disease, and dementia offer a unique opportunity for intervention because there are no disease-modifying agents for Alzheimer disease and related dementias [7]. More than 5.4 million people in the United States live with Alzheimer disease, the most common type of dementia [8]. Scientists predict the number of people with Alzheimer disease in the United States to reach 8.4 million by year 2030 [8]. Until disease-modifying agents are found, innovative psychosocial interventions, including mHealth interventions, offer the greatest potential for improving quality of life for persons with dementia and their caregivers [9].

Much remains unknown about the health outcomes used in mHealth apps and the effectiveness of these apps in improving the health of persons with MCI, Alzheimer disease, and dementia. There are literally hundreds of mobile apps that persons with MCI or dementia can use. Those mHealth apps are being marketed to help persons with cognitive impairment with unclear validity to their claims. Persons with cognitive impairment are already using mHealth apps, and will continue to do so in greater numbers, yet often the risks and benefits are not fully understood [3]. Similarly, the effects of these apps on persons with MCI, Alzheimer disease, and dementia have not been adequately reviewed and summarized in a systematic fashion. Therefore, the primary aim of this systematic review seeks to catalog the types of quantitative health outcomes utilized in these mHealth app studies. The secondary aim of this review strives to evaluate the effectiveness of mHealth apps in improving the health outcomes of persons with MCI, Alzheimer disease, and dementia through a review of the current scientific literature.

Background on the types of mHealth interventions included in this review are listed subsequently. These interventions can be grouped into a number of different categories, including cognitive training and serious games, wandering and wayfinding, reminiscence therapy, prompts and multicomponent interventions, engagement interventions, and exercise interventions.

### Types of Interventions

#### Cognitive Training and Serious Games

There exists a great deal of interest in using computerized cognitive training as an intervention to prevent and treat neurodegenerative disorders. Rebok et al [10] showed that independent older adults who underwent computerized cognitive training retained cognitive and functional benefits 10 years out from the intervention. However, the potential benefits for persons with MCI, Alzheimer disease, or persons with dementia is much less clear. A recent systematic review found that persons with MCI who received cognitive training had improvements in cognition, whereas persons with dementia had limited evidence for efficacy [11].

Serious games are games with a primary purpose other than entertainment, enjoyment, and fun [12]. They often include cognitive training or exercise training in the form of games. An example of this could be seen with a patient recovering from a stroke playing a serious game involving the activity of swinging a baseball bat in a virtual game rather than doing traditional exercises.

#### Wandering and Wayfinding

Wandering is a very common problem in older adults with mild-to-moderate stages of dementia. Such behavior usually occurs as a result of memory deficits and spatial disorientation, which makes persons with dementia less likely to recognize the route [13]. Navigation systems, such as satellite navigation (Global Positioning System), three-dimensional maps, and electronic maps, could provide assistance in locating the patient irrespective of the closed or outdoor environment, and could also support the person with dementia in finding their way back home [13,14]. This practice of finding one’s way back home or to a preselected destination is known as “wayfinding.”

#### Reminiscence Therapy

Reminiscence therapy works under the assumption that remote memory remains intact until later in the course of dementia and that recalling and discussing past events and life experiences can help the psychological wellness and cognition of people with dementia [15]. Often reminiscence therapy therapists will utilize music, pictures, art, and other aids in sessions. A therapist or a staff member trained in reminiscence therapy leads the session, which can take either a group or an individual therapy format. Reminiscence therapy has been shown to improve well-being, patient-caregiver relations, global cognition, and decrease social withdrawal [16-19].

#### Prompts and Multicomponent Interventions

With the progression of disease, persons with dementia lose their ability to perform activities of daily living (ADL) and often require frequent support and assistance from a family member or caregiver [20]. Prompts incorporate a unique approach to support and provide assistance to persons with cognitive impairment. Studies have shown that prompts can help persons with dementia to be less dependent on caregivers [21]. The function of prompts can range from reminders to take medications, to notifications of a scheduled activity, to a verbal or visual cue to get dressed or shower. We combined the prompt category and multicomponent intervention category because there was significant overlap between these two. Multicomponent interventions typically included prompts and some notification system for the caregiver. Other examples of components involve patient location, a communication system with health care professionals, and engagement activities.

#### Engagement Interventions

Past studies demonstrated that recreational activities and engagement can lead to persons with dementia having more positive affect, decreased agitation, and decreased passivity [22,23].

#### Exercise Intervention

Exercise training has been shown to reduce behavioral and psychological symptoms of dementia [24], to slow progression of cognitive decline in MCI [25], and to lead to increased hippocampus size [26], a region of the brain responsible for short-term memory. A recent review examining the evidence for physical and cognitive interventions to improve brain health found sufficient evidence that both physical and cognitive interventions lead to enhanced neuroplasticity and prevention of pathological aging (MCI, Alzheimer disease, and dementia) [27]. Evidence also suggests that the combination of physical and cognitive interventions may amplify these positive effects on neuroplasticity [27].

## Methods

Using the Preferred Reporting Items for Systematic Review and Meta-Analyses (PRISMA) checklist, we systematically reviewed the scientific literature to find mHealth apps that sought to improve health outcomes of persons with MCI, Alzheimer disease, and dementia. The PRISMA guidelines provide a standardized structure for the design, iterative process, extraction, and synthesis that take place during the development of a systematic review [28]. Aim 1 of this systematic review attempts to catalog quantitative health outcomes used to evaluate mHealth apps, whereas aim 2 seeks to assess the effectiveness of mHealth apps for persons with cognitive impairment that incorporate at least one quantitative health outcome. The systematic review protocol was registered with PROSPERO, the international prospective register of systematic reviews, at inception to avoid duplication [29].

### Data Sources and Searches

A comprehensive search of the literature was performed by a medical librarian (TWE) in Ovid, MEDLINE, PubMed, EMBASE, the full Cochrane Library, Cumulative Index to Nursing and Allied Health Literature (CINAHL), PsycINFO, Ei Compendex, IEEE Xplore, Applied Science & Technology Source, Scopus, Web of Science, ClinicalTrials.gov, and Google Scholar. All databases were searched from inception. Proceedings of the Annual Association for Computing Machinery (ACM) Conferences on Human Factors in Computing Systems, the ACM User Interface Software and Technology Symposium, and the IEEE International Symposium on Wearable Computers were searched in the ACM Digital Library from 2012 to 2016. Initial searches were conducted in February 2016 and updates were performed in May 2017. Bibliographies of relevant studies were also reviewed for additional references. A hand search of JMIR Publications journals was completed in July 2017. It should be noted that in the biomedical literature this type of separate search would be classified as a search of the “grey literature”; however, in the fields of computer science, engineering, and human computer interaction, conference proceedings are considered the primary source of scientific literature [30].

The complete search strategies for each database are reported in Multimedia Appendix 1. Database-specific subject headings and keyword variants for each of the two main concepts—dementia/cognitive impairment and mobile technology—were identified and combined. Results were limited to the English language, and animal studies were excluded.

### Study Eligibility

Only empirical studies were included in this systematic review. Inclusion criteria required the study to use a mHealth app on a tablet, a mobile phone, a personal digital assistant, another handheld mHealth device, or a mHealth app accessed from a computer as an intervention for persons with MCI, Alzheimer disease, or dementia. Studies using computers were only included if they accessed a program that was also a mHealth app.

Inclusion criteria required there be at least one quantitative health outcome in the study. Persons aged 18 years or younger were not included because the focus of this study was on cognitive impairment that develops during adulthood. Case series of more than two subjects, case-control studies, cross-sectional studies, and cohort studies were included. Studies were excluded if (1) the study focused primarily on participant populations outside of those with MCI, Alzheimer disease, and dementia. This included populations with diagnoses of traumatic brain injury, human immunodeficiency virus, multiple sclerosis, serious mental illness, intellectual disabilities, or active status as a caregiver, or (2) the primary purpose of the mHealth app was to screen for illness, make an assessment, or determine diagnosis. These studies were excluded because the focus of this study was on active mHealth interventions. Caregivers were not a target of this review. However, some studies included both persons with cognitive impairment and their caregivers. Studies were ruled out if caregivers were the population focus of the study.

### Study Selection and Data Extraction

Screening of records by title and abstract were completed independently by two authors (DB and BS). An adjudication process was used by the two authors, where they met face-to-face to review screened records. When the authors did not agree on a record, they came to a consensus together through discussion and re-review of the record. The same process was used when evaluating full articles for inclusion and when categorizing the final included articles with the Oxford Centre for Evidence-based Medicine (OCEBM) Levels of Evidence system. Both reviewers independently reviewed full articles and completed data extraction. Using a standard approach, they extracted study design, intervention type, technology type, population diagnoses, mean age of population, mean Mini-Mental Status Examination (MMSE) or comparable cognitive exam, health outcomes, and information on the effectiveness of the mHealth app. Study quality was assessed and categorized using the OCEBM Levels of Evidence System, where studies are categorized into one of five levels of evidence, with one being the strongest level [31]. Levels of evidence using the OCEBM system are (1) level 1: systematic reviews of randomized controlled trials (RCTs), individual RCTs, and all-or-none case series; (2) level 2: systematic reviews of cohort studies, individual cohort studies, and “outcomes” research; (3) level 3: systematic review of case-control studies and individual case-control studies; (4) level 4: case-series and poor quality cohort studies; and (5) level 5: expert opinion. Recommendation grades are listed as consistent level 1 studies (“A”), consistent level 2 or 3 studies or extrapolations from level 1 studies (“B”), level 4 studies or extrapolations from level 2 or 3 studies (“C”), and level 5 evidence or troubling inconsistent or inconclusive studies of any level (“D”) [31].

## Results

**Figure 1:**
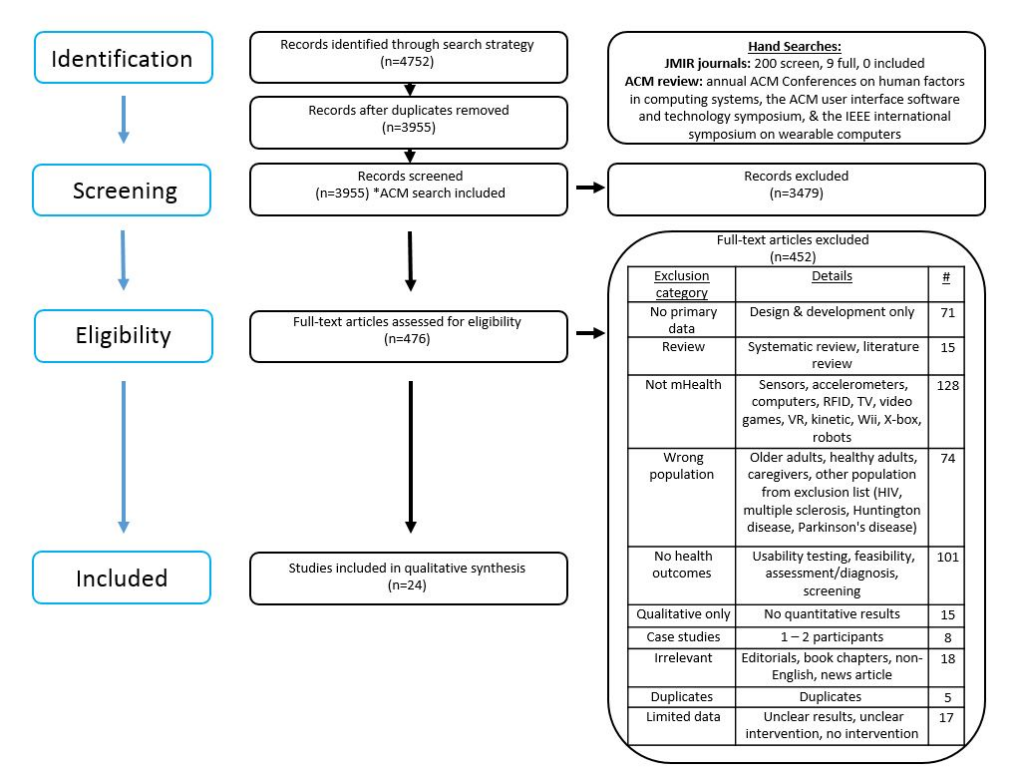
PRISMA flow diagram.

A total of 4752 records were identified through database searches. After removing duplicates, 3955 unique titles and abstracts were screened, and 476 full articles were reviewed (Figure 1) [32]. Division of these records according to database can be found in Table 1. A total of 24 separate articles met study inclusion criteria.

**Table 1:** Search results with and without duplicates.

Database	Duplicates included, n	Duplicates removed, n
ACM Digital Library	292	289
Applied Science &Technology Source	37	23
CINAHL	360	291
ClinicalTrials.gov	47	47
Cochrane Library	224	105
Ei Compendex	120	106
EMBASE	736	640
Google Scholar	67	37
IEEE Xplore	453	344
Ovid MEDLINE	1277	1276
PsycINFO	250	134
PubMed	507	507
Scopus	207	106
Web of Science	175	50
Total	4752	3955

Health outcomes were extracted and grouped by intervention type (Table 2). Of the 24 individual studies included, 14 studies lacked controls. The majority of studies were small in size. Using the Modified OCEBM Levels of Evidence rating system, four studies met criteria for level 2 evidence [33-36] and none met criteria for either level 1 or level 3 evidence.

The remaining 83% (20/24) studies were identified as meeting criteria for level 4 evidence (Tables 3-6). All studies were found in biomedical journals or were from biomedical conferences. None of the included studies came from the engineering literature. Two of the four level 2 studies showed some evidence of efficacy [33,39]. When looking at all 24 studies regardless of quality, 58% (14/24) showed some degree of efficacy.

**Table 2:** Health outcomes and efficacy by mHealth intervention type (N=24).

Intervention type	Studies, n^a^	Health outcomes	Studies with efficacy, n (%)
Cognitive training with no games	6	Cognition [33-35,37-39]; function [35]; mood [33]	5 (83)
Serious games	4	Cognition [36,40-42]; mood, anxiety, stress [36]	1 (25)
Wandering and wayfinding	1	Cognition [43]; unsafe walking behavior [43]	1 (100)
Reminiscence therapy	2	Cognition [19]; communication ability [19]; mood [19]; social interest [19]; psychological stability [44]	2 (100)
Prompts and multicomponent interventions	4	Cognition [20,45]; subjective report of cognition [45]; mood [45]; psychological stability [44]; perceived autonomy [20,46]; feeling of competence [20]; number of caregiver and patient unmet needs [20]; quality of life for caregiver or patient [20,45,46]; caregiver burden [45]	1 (25)
Engagement interventions	7	Cognition [47]; well-being and mood [47-49]; behavioral and psychological symptoms of dementia [50-52]; engagement in activities [47,48]; quality of life of patient [48]; helpfulness to caregiver [53]	5 (71)
Exercise intervention	1	Quality of life, self-efficacy, change in weekly steps taken, 6-min walk, Mini-Physical Performance Test [54]	0 (0)
Total	24		14 (58)

^a^Categories are not mutually exclusive; one article was counted twice.

**Table 3:** Cognitive training interventions (n=6).

Author, year	Study type	Intervention type	Technology type	Population^a^	Outcomes	OCEBM level^b^	Effective/Results^c^
Barnes et al, 2006 [33]	RCT	Cognitive training	Computer	36 pts w/ MCI; age: mean 74 years; RBANS total: mean 86.6	Cognition, mood	2	No improvement in cognition (RBANS total)
Chan et al, 2017 [34]	RCT-single blind	Cognitive training	Tablet	99 pts w/ MCI; age: mean 68.7 years; MOCA: mean 24.4	Cognition	2	Yes, treatment group had improvements in working memory; both treatment and control groups had improvements in immediate and delayed recall
Gooding et al, 2015 [37]	RCT	Cognitive training	Computer	74 pts w/ subclinical cognitive decline; age: mean 75.6 years; mMMSE: mean 50.6	Cognition	4	Yes, improvement in cognition (mMMSE, BSRT, and LMS)
Han et al, 2014 [38]	Pilot	Cognitive training	Tablet	10 pts w/ MCI; age mean: 69.7 years; MMSE: mean 26.7; CDR: mean 0.5	Cognition	4	Yes, significant improvement in cognition (word list memory test)
Mansbach et al, 2017 [39]	Controlled trial	Cognitive training	Mobile app from computer	38 pts w/ normal cognition, MCI, and mild dementia; age: mean 78.1 years; BCAT: mean 37.3	Cognition, subjective report of cognition	4	Yes, treatment group had greater improvements in cognition
Tarraga et al, 2006 [35]	RCT	Cognitive training	Computer	43 pts w/ MCI; age: mean 76.7 years; MMSE: mean 21.9	Cognition, function	2	Yes, improvement in cognition (ADAS-Cog, MMSE); no functional improvements

^a^BCAT: Brief Cognitive Assessment Tool; CDR: Clinical Dementia Rating scale; MCI: mild cognitive impairment: MMSE: Mini-Mental State Examination; mMMSE: Modified Mini-Mental State Examination; MOCA: Montreal Cognitive Assessment.

^b^Oxford Centre for Evidence-based Medicine’s Levels of Evidence and Grades of Recommendation (1=highest quality; 5=lowest quality).

^c^ADAS-Cog: Alzheimer’s disease Assessment Scale cognitive subscale; BSRT: Buschke Selective Reminding Test; LMS: Logical Memory Subtest; RBANS: Repeatable Battery for the Assessment of Neuropsychological Status.

**Table 4:** Serious games with cognitive training (n=4).

Author, year	Study type	Intervention type	Technology type	Population^a^	Outcomes	OCEBM level^b^	Effective/Results
Finn and McDonald, 2011 [36]	RCT	Serious games, cognitive training	Computer	25 pts w/ MCI; age: mean 74.2 years; MMSE: mean 27.8	Cognition, Mood, Anxiety, Stress	2	No, only improvement was in visual sustained attention; no improvement in cognition, depression, or anxiety
Hsiung et al, 2009 [40]	Pilot	Serious games, cognitive training	Handheld device	17 pts total 12 w/ MCI, 2 healthy, 3 w/ subjective memory complaints; age: mean 72 years: MMSE: mean NR	Cognition	4	No improvement in cognition
Manera et al, 2015 [41]	Pilot	Serious games, cognitive training	Tablet	9 pts w/ MCI, 12 pts w/ Alzheimer disease; age mean 78.4 years; MMSE MCI: mean 27.2, MMSE Alzheimer disease: mean 18.4	Cognition	4	Yes, improvement in praxis & executive function
Merilampi et al, 2014 [42]	Pilot	Serious games, cognitive training	Tablet, computer	16 pts w/ mild-to-moderate cognitive impairment; age: mean 90 years; MMSE: mean 21.6	Cognition	4	No improvement in cognition

^a^MCI: mild cognitive impairment; MMSE: Mini-Mental State Examination; NR: not reported.

^b^Oxford Centre for Evidence-based Medicine’s Levels of Evidence and Grades of Recommendation (1=highest quality; 5=lowest quality).

**Table 5:** Wandering and wayfinding (n=1), reminiscence therapy (n=2), and prompts and multicomponent (n=4) interventions.

Author, year	Study type	Intervention type	Technology type	Population^a^	Outcomes^b^	OCEBM level^c^	Effective/Results^b^
Hettinga et al, 2009 [43]	Pilot	Wandering and wayfinding	PDA	4 pts w/ mild dementia; age: ≥55 years; MMSE: range 17-25	Unsafe walking behaviors, working memory	4	No unsafe walking behaviors
Hattink et al, 2016 [20]	RCT	Multicomponent intervention	Early detection system-touchscreen or mobile device	42 pts w/ MCI and dementia; age: mean 78.7 years; MMSE: mean 18.1	Cognition, QOL-AD for CG or patient, perceived autonomy, feeling of competence, number of CG and patient unmet needs	4	No differences in QOL-AD for CG or patient, perceived autonomy, grade for QOL, feeling of competence, MMSE, number of caregiver and patient unmet needs.
Imbeault et al, 2016 [45]	Case series	Prompts	Mobile phone app	3 pts w/ Alzheimer disease; age: mean 69 years; MMSE: mean 28	Cognition, subjective report of cognition, depression, CG burden	4	No, almost all cognitive tests remained the same or decreased. Unclear results for depression and caregiver burden.
Meiland et al, 2012 [46]	Pilot	Prompts	Mobile device, sensors, touchscreen, & actuators	12 pts w/ MCI, dementia, or Alzheimer disease; age: range 57-84 years; MMSE: range 17-25 (mean age and MMSE NR)	Quality of life, perceived autonomy	4	No effect on QOL of Patient or CG, or perceived autonomy.
Yasuda et al, 2013 [44]	Pilot	Prompts and reminiscence therapy	Computer	4 pts w/ Alzheimer disease; age: mean 78.7 years; MMSE: mean 19.5	Psychological stability, communication ability, IADL completion	4	Yes, 3 of 4 participants had benefit in psychological stability. Improvements were also noted in communication ability and in IADL competition.
O’Rourke et al, 2011^d^ [19]	Pilot	Reminiscence therapy	Mobile app from computer	6 pts w/ dementia; age: mean 72 years; MMSE: mean 17.8	Cognition, communication ability (FLCI), depression, social interest questionnaire	4	Yes, MMSE scores improved in half of pts FLCI scores improved or remained stable in all but one participant.

^a^MCI: mild cognitive impairment; MMSE: Mini-Mental State Examination; NR: not reported.

^b^CG: caregiver; FLCI: Functional Linguistic Communication Inventory; IADL: Instrumental Activities of Daily Living; QOL-AD: Quality of Life Scale in Alzheimer’s Disease.

^c^Oxford Centre for Evidence-based Medicine’s Levels of Evidence and Grades of Recommendation (1=highest quality; 5=lowest quality).

^d^Categories are not mutually exclusive; one article was counted in both the reminiscence therapy and prompts section.

**Table 6:** Engagement (n=7) and exercise (n=1) interventions.

Author, year	Study type	Intervention type	Technology type	Population^a^	Outcomes^b^	OCEBM level^c^	Effective/Results
Astell et al, 2016 [49]	Controlled trial	Engagement	Tablet	30 pts w/ dementia; age: mean 87.3 years; MOCA: mean 13.4	Enjoyment	4	Yes, 88% of patients reportedly enjoyed the games
Hsu et al, 2016 [50]	Case series	Engagement	Tablet	3 pts w/ dementia; age: mean 78 years; MOCA: mean 23.5 (1 pt refused MOCA)	Behavioral and psychological symptoms of dementia	4	Yes, decreased use of “as-needed” medications for behavioral problems
Leng et al, 2014 [47]	Pilot	Engagement	Tablet	6 pts w/ dementia; age: mean 77 years; MMSE: mean 21	Cognition, mood, engagement, well-being	4	Yes, tablet activities at least an equal positive effect on mood, engagement, and well-being vs traditional group activities
Lim et al, 2013 [53]	Pilot	Engagement	Tablet	21 dyads of people with early dementia and CGs; PWD age: mean 73.5 years; MMSE: NR	Helpfulness to caregiver	4	Yes, 47.6% of CG found tablet somewhat, moderately, or extremely helpful
Tyack et al, 2015 [48]	Pilot	Engagement	Tablet	12 dyads-PWD and CGs; age: mean 75 years; MMSE: NR	QOL-AD; Visual Analogue Scale for Happiness, Wellness, and interestedness	4	No improvement in happiness, wellness, interestedness
Vahia et al, 2016 [51]	Open-label study	Engagement	Tablet	36 pts w/ dementia; age: mean 79.9 years; MMSE: NR	Behavioral and psychological symptoms of dementia, agitation	4	Yes, agitation decreased post intervention
Van Der Ploeg et al, 2015 [52]	RCT	Engagement	Mobile app from computer	17 pts w/ dementia; age: mean 86.7 years; MMSE: mean 7.3	Behavioral and psychological symptoms of dementia, agitation	4	No significant reduction in agitation
Vidoni et al, 2016 [54]	Pilot	Exercise	Accelerometer, mobile app from computer	21 pts as normal control, 9 pt w/ Alzheimer disease; control age: mean 72.3 years, Alzheimer disease age: mean 69.6 years, MMSE: NR	QOL, self-efficacy, change in weekly steps taken, 6-min walk, Mini-Physical Performance Test	4	No improvement in outcomes

^a^CG: caregiver; MMSE: Mini-Mental State Examination; MOCA: Montreal Cognitive Assessment; NR: not reported; PWD: people with dementia.

^b^QOL: Quality of Life; QOL-AD: Quality of Life Scale in Alzheimer’s Disease.

^c^Oxford Centre for Evidence-based Medicine’s Levels of Evidence and Grades of Recommendation (1=highest quality; 5=lowest quality).

### Cognitive Training and Serious Games

#### Study Characteristics

Cognitive training with and without serious games made up a large number of the studies in this review (n=10). Intervention design and duration varied significantly among studies. All studies in the two groups incorporated cognition as an outcomes measure, another included function [35] and still others used mood, stress, and anxiety [33,36] as outcomes. When examining the two groups in combination 60% (6/10) of the studies showed some degree of efficacy [34,35,37-39,41] (see Tables 3 and 4). All four studies in this review that met criteria for level 2 quality of evidence fell into one of these two categories [33-36].

#### Description of Apps and Technology

A number of studies in this group used commercially available cognitive training programs, such as those by the company Lumos Laboratory, marketed as Lumosity, and by the company Posit Science marketed as BrainHQ. Other studies reported on mHealth apps that had been developed through research. Many of these studies were conducted on computers; however, studies were only included if the apps were able to be accessed by mobile devices. One study that demonstrated efficacy used a tablet-based Chinese calligraphy program as a form of cognitive training [34].

### Wayfinding and Wandering

#### Study Characteristics

Although a number of pilot studies reported on experiments involving navigation systems for persons with dementia, only one met criteria for inclusion [43]. Health outcomes for the study consisted of working memory and unsafe walking behaviors [43]. The study proved to be effective in that no unsafe walking behaviors were found for those who used the navigation system.

#### Description of Apps and Technology

In the study by Hettinga et al [43], software called TomTom was used for navigation support. They studied the safety of TomTom use by people with dementia and the effectiveness of familiar versus unfamiliar voice prompts. The use of navigation software was found to be safe based on observations of street-crossing behavior, response to navigation instructions, and number of occurrences of stopping at device prompts. Additionally, they observed that familiar voice prompts were more effective compared to unfamiliar ones. This was determined by measuring walking time, number of errors (route deviations and repeated instructions), and number of times assistance was requested. Warning sounds seemed to have a negative effect on wayfinding [43]. Participants were captured on video. These videos were coded for unsafe walking behaviors [43].

### Reminiscence Therapy

#### Study Characteristics

Two studies incorporated a reminiscence therapy intervention [19,44]. Health outcomes included cognition, communication ability, mood, social interest, and psychological stability. Both studies showed some degree of efficacy [19].

#### Description of Apps and Technology

O’Rourke et al [19] used the Web-based video website YouTube to help facilitate reminiscence therapy in persons with dementia. Five of six participants showed improvement or stability in their communication ability over the 6-week pilot study. They concluded that the website YouTube is a suitable tool for delivering personalized computer-based reminiscence therapy [19]. YouTube also functions as a mobile app.

In their pilot study, Yasuda et al [44] used a videophone as both a remote reminiscence conversation system and as a schedule prompter system. The remote reminiscence conversation system shared reminiscence photos through a videophone triggered by the conversation partner on the other end of the phone. The study results showed following conversations with the system patients had improved psychological stability, verbal communication, and rates of instrumental ADL completion. The schedule prompter system used more than 10 different video reminders, such as prompts to take medications or to prepare meals. Individual experiments were conducted to evaluate each system using four patients with dementia. The first experiment evaluated the effectiveness of the remote reminiscence conversation system by performing two tasks: watching TV and remote video chatting. Results were measured using the Gottfries-Brane-Steen (GBS) scale that measures psychological variables such as confusion, irritability, anxiety, restlessness, reduced mood, and agony. The GBS scale is scored on a scale from zero (most stable) to six (least stable). Results showed that three of four patients obtained psychological stability as defined by the authors. The second experiment determined the effectiveness of a schedule prompter system in completing the scheduled task. Participants received three different types of video prompts: navigational prompts to move toward the computer, motivational prompts to inspire completion of tasks, and scheduled prompts to remind participants of tasks scheduled for completion. The results described the mean completion of tasks for the four patients to as 83% while using the prompter system [44].

### Prompts and Multicomponent Interventions

#### Study Characteristics

We found four studies in these combined categories [20,44-46]. Health outcomes for this group included cognition, subjective report of cognition, mood, psychological stability, perceived autonomy, feeling of competence, the number of caregiver and patient unmet needs, caregiver burden, and the quality of life for the caregiver and patient. Of the four studies in this category, only the study by Yasuda et al [44] showed improvement in health outcomes [44].

#### Description of Apps and Technology

Yasuda et al [44] was previously described in the reminiscence therapy section because the study contained both reminiscence therapy and prompt components. Overall, in these studies, prompts were delivered in either an auditory or visual manner. The use of mobile devices in this group varied. Imbeault et al [45] studied the impact of an electronic organizer “AP@LZ” on the cognition, subjective report of cognition, depression, and caregiver burden of three persons with Alzheimer disease. Unfortunately, there were no cognitive benefits. The results of the effect on depression and caregiver burden were unclear [45].

In 2009, Meiland et al [46] evaluated the use of a digital prosthetic, “COGNOW Day Navigator,” by 12 persons with dementia and their caregivers. The digital prosthetic was designed to help with memory, social contacts, daily activities, and safety. Participants rated the study as useful and user-friendly. Effectiveness of the system could not be determined because of the short study duration and instability of the digital prosthetic prototype.

Lastly, one multicomponent intervention study by Hattink et al [20] met criteria for inclusion. This intervention, “Rosetta,” targeted four domains of required support for persons with dementia: (1) prompts and reminders, (2) leisure, (3) communication, and (4) safety. The Rosetta intervention was tested with 42 patients with either MCI or Alzheimer disease. Contained in their measured health outcomes were cognition, perceived autonomy, feelings of competence, the number of unmet caregiver and patient needs, and quality of life of the caregiver and patient. No improvements in outcomes occurred following the intervention [20].

### Engagement Interventions

#### Study Characteristics

Past studies demonstrated that recreational activities and engagement can lead to persons with dementia having more positive affect, decreased agitation, and decreased passivity [22,23]. Health outcomes varied by study. They consisted of mood, engagement, well-being, behavioral and psychological symptoms of dementia, agitation, and quality of life of the caregiver and patient. Five of the seven (71%) studies using a mHealth app intervention to engage people with dementia in activities had evidence of efficacy [47-53].

#### Description of Apps and Technology

Tablets were the technology of choice for these studies. In an adult day program, Leng et al [47] studied how iPad group activity sessions compared to traditional group engagement activities of cooking and arts and crafts for persons with dementia. The study found that for the persons with dementia who participated, iPad activities had at least an equal positive effect on mood, engagement, and well-being as compared to traditional group activities [47].

Another group, Lim et al [53], studied the usability of iPads by persons with dementia and their caregivers. In all, 95% of persons with dementia participating in the study had not previously used an iPad. Apps in the categories of art, music, simple interactive games, and relaxation were loaded onto each iPad. The patient-caregiver dyad was given the iPad to take home and use for 7 days with the recommendation that the caregiver provide 30 minutes of daily supervision and interaction while the person with dementia used the iPad. Nearly half of the caregivers indicated the iPad was somewhat, moderately, or extremely helpful.

Astell et al [49] studied 30 persons with dementia and measured the impact familiar and nonfamiliar games on a tablet had on the participants’ enjoyment. A total of 90% of participants attempted to use the tablet. Regardless of familiarity, close to 90% of participants displayed enjoyment from playing games on a tablet.

Tyack et al [48] developed and tested an art viewing tablet app for persons with dementia as a well-being intervention. Twelve patient-caregiver dyads participated. Participants were asked to use the tablet and program five times over a 2-week span and were given a list of questions to facilitate conversation while using the app. There were no significant pre-post differences in any of the outcomes measured, including patient happiness, wellness, engagement, and patient and caregiver quality of life [48]. Despite this, there was a trend toward improvement in all outcome categories, indicating that a larger study might demonstrate effect.

Van der Ploeg et al [52] tested Internet video conferencing (Skype) and telephone calls with family members as an intervention to reduce agitation in persons with dementia. Preintervention and postintervention measurements showed no difference in Cohen-Mansfield Agitation Inventory scores [52].

Vahia et al [51] and Hsu et al [51] both used mobile apps on a tablet to treat symptoms of agitation and behavioral and psychological symptoms of dementia. Vahia et al found that their intervention reduced agitation and that all participants including those with severe dementia were able to use apps on the tablet [51]. In the small study by Hsu et al, those who received the tablet intervention had decreased use of “as-needed” medications to treat behavioral and psychological symptoms of dementia [50].

### Exercise Intervention

#### Study Characteristics

The one exercise intervention that met inclusion criteria selected health outcomes of quality of life, self-efficacy, change in weekly steps taken, the 6-minute walk time, and the Mini-Physical Performance Test. None of these outcomes improved with the exercise intervention [54].

#### Description of App and Technology

Vidoni et al [54] sought to improve the health of persons with dementia by prescribing physical activity in conjunction with using a wearable mHealth device (Fitbit) in a tertiary medical clinic setting. The wearable device was an accelerometer that measured steps taken and communicated with the mHealth app installed on either a mobile device or computer. The randomized trial included participants with normal cognition and Alzheimer disease-related cognitive impairment. The study was designed to last 16 weeks, but only 8 weeks of data was reported. Normal controls improved on their baseline weekly steps taken, whereas those with cognitive impairment did not. Only 62% of those with cognitive impairment completed the intervention [54].

## Discussion

### Theoretical Implications

To our knowledge, this comprehensive review is the first to examine the efficacy of mHealth app interventions on the health outcomes of persons with MCI, Alzheimer disease, and dementia. We employed state-of-the-art methodology in identifying the relevant literature, rating the quality of studies, and extracting standardized data. Using a broad search strategy, we discovered this literature spread across the research and innovation outlets of multiple disciplines. The level of evidence supporting the use of mHealth app interventions for people with these disorders (MCI, Alzheimer disease, and dementia) was low, as reflected by a grade C level of evidence using the modified OCEBM rating system. Some degree of efficacy was seen in 58% (14/24) of all included studies. However, given the limited quality of these studies it is difficult to draw any firm conclusions. Given the potential size of the market for these interventions and their apparent potential for improving the care of persons with cognitive impairment, we uncovered a need not only for more research in this area, but also for greater agreement in study design and consensus on health outcome measures.

Unexpectedly, none of the 24 studies came from the primary computer science literature. We attribute this lack of computer science study selection to the inclusion criteria requiring at least one quantitative health outcome. In our review, we found a number of innovative studies of mHealth app interventions for people with cognitive impairment. In the computer science literature, however, the vast majority of these studies lacked any measure of patient health. This significant finding highlights the potential opportunity and need for collaboration between technology researchers and health care professionals to develop mHealth app interventions that improve the health of individuals with cognitive impairment.

### Outcomes

Aim 1 was to examine and catalog the types of quantitative health outcomes used by mHealth app interventions for persons with MCI, Alzheimer disease, and dementia. We found that 101 of 476 (21.2%) articles fully reviewed were excluded because of a lack of health outcomes. There were also inconsistencies in the health outcomes selected by study investigators. There appears to be a lack of consensus on which health care outcomes should be used to evaluate mHealth app interventions targeting those with cognitive impairment. The most commonly used health outcomes were cognition, function, quality of life, mood and well-being, and behavioral and psychological symptoms of dementia. There exists a greater need for consistency in the health outcomes for persons with cognitive impairment in these studies.

### Efficacy

Aim 2 of this study sought to determine the efficacy of mHealth app interventions focused on persons with cognitive impairment that included at least one quantitative health outcome in the study evaluation. This systematic review found that currently there is little evidence to support the efficacy of most mHealth app interventions improving the health of persons with cognitive impairment. The number of mHealth apps continues to grow [3]. However, there is limited oversight and rigor in the development and testing of these apps and their claims of benefit. Lumos Laboratory, doing business as Lumosity, served as the most recent well-publicized example of a company charged with unproven medical claims about the benefits received from their cognitive training mHealth app. The Federal Trade Commission alleged that the company misled consumers in claiming that their product “delays age-related mental decline and protects against dementia and Alzheimer’s disease” [55]. Without rigorous RCTs to prove efficacy of individual mHealth app interventions, mHealth apps run the risk of being the newest “snake oil” to treat dementias and associated disorders. More work needs to be done to determine which apps are effective at improving patient outcomes.

### Limitations

Our review has several limitations. The review topic was broad, which creates challenges in terms of in-depth data synthesis. An adjudication process between two authors (DB and BS) was used to find consensus on article screening, full article review, and categorization with the OCEBM Levels of Evidence System. Interrater reliability or kappa was not calculated and is therefore a limitation. This review’s original search strategy did not focus explicitly on cognitive training; therefore, there may have been studies in this area that were not captured in this review. In the field of mHealth and in our review the majority of the studies are of small size and lacked sufficient quality in study design.

### Future Directions

Greater need for clinical trials to test mHealth interventions necessitates involvement with the Food and Drug Administration (FDA), the government agency with the widest jurisdiction over the regulation of mobile health technologies [56]. The FDA issued its most recent nonbinding industry and staff guidance document on “mobile medical applications,” in 2013 and updated the document in 2015 [57]. In these reports, The FDA defines mobile medical apps as “a mobile app that meets the definition of device in section 201(h) of the Federal Food, Drug and Cosmetic Act (FD&C Act); and is either intended (1) to be used as an accessory to a regulated medical device; or (2) to transform a mobile platform into a regulated medical device” [57]. Section 201 (h) describes a medical device as one “...intended for use in the diagnosis of disease or other conditions, or in the cure, mitigation, treatment, or prevention of disease, in man...” or “...intended to affect the structure or any function of the body of man...” This definition includes software or apps on computers, websites, and handheld devices.

The FDA has made clear their intention to apply regulatory oversight to the subset of mobile medical apps that transform a mobile platform into a medical device [56,57]. They indicate that they reserve the right to enforce the guidelines at their discretion and will focus on mobile medical apps that have the potential to cause harm to patients [57].

Outside of efficacy and safety, other concerns exist with mHealth technologies, including privacy risks. In their 2016 *JAMA* letter, Blenner et al [58] showed that 81% of available Android diabetes apps did not have privacy policies and that 48.4% of diabetes apps with privacy policies shared user information. In downloading and installing an app, health consumers often inadvertently give apps permission to collect personal information [58]. As noted by Blenner et al, there are currently no federal laws to prevent app companies from selling medical app data to third parties [58,59]. One could imagine the potentially damaging effect that could be incurred if sensitive medical information were to be shared with health or life insurance companies or with a prospective employer.

Although not the focus of this review, we found a large number of studies did not take end users’ (persons with dementia or caregivers) input into consideration during the development of mHealth interventions, creating potential mismatch between the proposed solution and the participant needs. This suggests an opportunity for greater emphasis on user-centered design. User-centered design is a philosophy and methodology for designing and evaluating systems based on end-user involvement and a strong understanding of end-user characteristics, goals, tasks, needs, capabilities, and contexts [60]. This approach has the ultimate goal of optimal functioning of the human-machine system [61].

Despite these different concerns and drawbacks, mHealth apps possess great potential to improve the health and outcomes of people with cognitive impairment and offer advantages over traditional psychosocial interventions. With the growing numbers of persons with dementia, limitations of family caregivers, and work force shortage, there is an extraordinary need for engagement and social support of persons with dementia. One could imagine mHealth apps that could engage persons with dementia and help improve their mood through serious games or through a mHealth peer-support program. The aim of these apps would not be to replace caregivers or humans, but rather to allow for engagement when caregivers or others are busy or not immediately available.

The use of mHealth apps offer an opportunity to help persons with MCI, Alzheimer disease, and dementia maintain their independence longer than would be otherwise possible. Apps designed to prompt persons with dementia with necessary tasks such as taking medication, taking out the garbage, or cooking meals, could help persons with dementia complete these essential tasks rather than having to hire a caregiver or move into an assisted living to receive support. Similarly, wayfinding apps could improve persons with dementia’s ability to travel safely in their community. And remote monitoring apps could allow caregivers and health care providers to supervise or receive notifications of changes in the health status of the person with dementia.

In addition, mHealth apps could improve measurement accuracy of variables pertaining to the health status of persons with dementia. Through ecological momentary assessment, the repeated sampling of a participant’s experience or behaviors in real time, an app could provide up-to-date information on a person’s mood status or neuropsychiatric symptoms of dementia [62], thus avoiding the pitfalls of recall bias seen with more traditional office-based questionnaires. Monitoring or prompting apps could help measure real-time decline in a person with dementia’s cognition or function, offering providers a more accurate depiction of disease progression.

Virtual coaches hold promise as the next generation of mHealth apps and have the potential to help persons with cognitive impairment. Siewiorek et al [63] from Carnegie Mellon University and the National Science Foundation-funded Quality of Life Technology Center have pioneered the theory and design of virtual coaches [63,64]. A virtual coach moves beyond the rote and static reminders of a prompt system. Rather, a virtual coach adequately adapts to the needs of the user. The ideal qualities of a virtual coach as a cognitive aid, as outlined by Siewiorek et al [63], include the virtual coach reducing the number of cues as the user learns, matching the level of support to changes in the user’s ability, allowing for caregivers to upload new capabilities to the virtual coach and providing consistent monitoring of adherence to a caregiver’s instructions.

### Conclusion

We found that many mHealth app interventions targeting those with cognitive impairment lack health outcomes as a part of their evaluation process and that there is a lack of consensus as to which health outcomes should be used. Of note, the most common health outcomes in this review were cognition, function, quality of life, mood and well-being, and behavioral and psychological symptoms of dementia. These align with outcomes for clinical trials for Alzheimer disease as described in previous systematic reviews [65-67]. From their comprehensive review of the scientific literature in 2017, Bentvelzen et al [67] distilled a list of best practice outcomes for dementia, called the Dementia Outcome Measurement Suite. Our results suggest that a best practice or at least greater consensus is needed around selection of appropriate health outcomes for mHealth app interventions targeting persons with cognitive impairment. The Dementia Outcome Measurement Suite is one best practice guideline that could be considered. The suite has six outcome domains: (1) cognition, (2) staging, (3) function, (4) behavior, (5) delirium, and (6) quality of life [67]. More in-depth discussion of these domains lies outside the scope of this review.

The evidence that use of mHealth app interventions improves the health of people with MCI, Alzheimer disease, and dementia is of limited quality. Evidence met criteria for grade C level of quality as per the OCEBM Levels of Evidence System. Study reports of efficacy were mixed with more than half of the studies (58%) showing some degree of effectiveness. More RCTs, a larger number of participants, and a design that minimizes bias are needed to better clarify the benefits of these types of interventions.

## Appendix

Multimedia Appendix 1 Search Strategy.

## Abbreviations

ADL: activities of daily living
FDA: Food and Drug Administration
GBS: Gottfries-Brane-Steen
MCI: mild cognitive impairment
MMSE: Mini-Mental Status Examination
NIH: National Institutes of Health
OCEBM: Oxford Centre for Evidence-based Medicine
PRISMA: Preferred Reporting Items for Systematic Review and Meta-Analyses
RCT: randomized controlled trials
