# Supplementary material for: Categorizing Health Outcomes and Efficacy of mHealth Apps for Persons With Cognitive Impairment: A Systematic Review
Source: J Med Internet Res. 2017 Aug 30;19(8):e301. doi: 10.2196/jmir.7814 (PMC5597798; doi:10.2196/jmir.7814)
Supplement: Multimedia Appendix 1 [file jmir_v19i8e301_app1.pdf]

Supplement 1: Detailed Search Strategies

MEDLINE (Ovid):

1	exp Dementia/
2	exp Cognition Disorders/
3	(alzheimer* or dementia*).mp.
4	(cognitive impairment or neurocognitive disorder* or neuro cognitive disorder*).mp.
5	exp Mobile Applications/
6	exp Cell Phones/
7	exp Computers, Handheld/
8	(smartphone* or smart phone* or personal digital).mp.
9	(iphone* or i phone* or ipad* or i pad*).mp.
10	((handheld or hand held) adj (comput* or device*)).mp.
11	(android or itune* or google play or ios).mp.
12	(mobile adj2 (phone* or device* or app$1 or application* or technolog*)).mp.
13	((laptop* or tablet) adj2 computer*).mp.
14	(mhealth or m health or mobile health).mp.
15	("information and communication* technolog*" or ict).mp.
16	(assistive technolog* or information technolog*).mp.
17	exp Self-Help Devices/
18	exp Videoconferencing/
19	exp Computer Literacy/
20	exp Computer Communication Networks/
21	exp Software Design/
22	exp User-Computer Interface/
23	exp Video Games/
24	exp Web Browser/
25	exp Wireless Technology/
26	Therapy, Computer-Assisted/
27	Computers/
28	Technology/
29	(or/1-4) and (or/5-28)
30	limit 29 to english language
31	exp Animals/ not exp Humans/
32	30 not 31

PubMed (PubMed.gov):

#1	alzheimer* [tiab] OR dementia* [tiab] OR “cognitive impairment” [tiab] OR ((neurocognitive [tiab] OR “neuro cognitive” [tiab]) AND disorder* [tiab])
#2	(mobile [tiab] AND (phone* [tiab] OR device* [tiab] OR app [tiab] OR apps [tiab] OR application* [tiab] OR technolog* [tiab])) OR ((cell [tiab] OR cellular [tiab] OR smart [tiab]) AND (phone* [tiab] OR telephone* [tiab])) OR smartphone* [tiab] OR handheld [tiab] OR “hand held” [tiab] OR

“personal digital” [tiab] OR iphone* [tiab] OR i phone* [tiab] OR ipad* [tiab] OR i pad* [tiab] OR android [tiab] OR itune* [tiab] OR google play [tiab] OR ios [tiab] OR wireless* [tiab] OR mhealth [tiab] OR “m health” [tiab] OR communication technolog* [tiab] OR communications technolog* [tiab] OR assistive technolog* [tiab] OR information technolog* [tiab] OR ict [tiab] OR “self help” [tiab] OR podcast* [tiab] OR videos [tiab] OR videoconferenc* [tiab] OR videogame* [tiab] OR videophone* [tiab] OR game [tiab] OR games [tiab] OR internet [ti] OR web [ti] OR computer* [ti] OR technolog* [ti]

#3 ((#1 AND #2) NOT medline [sb]) Filters: English

Embase (Embase.com)

#1 'dementia'/exp/mj

#2 'dementia assessment'/exp/mj

#3 'mild cognitive impairment'/exp/mj

#4 alzheimer*:ti OR dementia*:ti OR 'cognitive impairment':ti OR neurocognitive:ti OR 'neuro cognitive':ti

#5 'mobile application'/exp

#6 'communication software'/exp

#7 'web browser'/exp

#8 'mobile phone'/exp

#9 'microcomputer'/exp

#10 'self help device'/exp

#11 'social media'/exp

#12 'text messaging'/exp

#13 'webcast'/exp

#14 'wireless communication'/exp

#15 'human computer interaction'/exp

#16 'computer network'/exp

#17 'video game'/exp

#18 'internet'/exp

#19 'computer assisted therapy'/de

#20 'personal digital assistant'/exp

#21 'computer'/de

#22 smartphone*:ti OR (smart NEXT/1 phone*):ti OR 'personal digital':ti OR iphone*:ti OR (i NEXT/1 phone*):ti OR ipad*:ti OR (i NEXT/1 pad*):ti OR ((handheld OR 'hand held') NEXT/1 (comput* OR device*)):ti OR android*:ti OR itune*:ti OR 'google play':ti OR ios:ti

#23 mobile:ti AND (phone*:ti OR device*:ti OR app:ti OR apps:ti OR application*:ti OR technolog*:ti)

#24 computer*:ti OR software:ti

#25 mhealth:ti OR 'm health':ti OR 'mobile health':ti OR ehealth:ti OR 'e health':ti

#26 (information:ti AND communication*:ti AND technolog*:ti) OR ict:ti

#27 (assistive NEXT/1 technolog*):ti OR (information NEXT/1 technolog*):ti

#28 (#1 OR #2 OR #3 OR #4) AND (#5 OR #6 OR #7 OR #8 OR #9 OR #10 OR #11 OR #12 OR #13 OR #14 OR #15 OR #16 OR #17 OR #18 OR #19 OR #20 OR #21 OR #22 OR #23 OR #24 OR #25 OR #26 OR #27)

#29 'animal'/exp NOT 'human'/exp

#30 (#28 NOT #29) AND [english]/lim

Cochrane Library (Wiley):

#1 (alzheimer* or dementia* or "cognitive impairment" or neurocognitive or "neuro cognitive"):ti,ab
#2 (mobile or app or apps or ((cell or cellular or smart) next (phone* or telephone*)) or smartphone* or handheld or "hand held" or "personal digital" or iphone* or i next phone* or ipad* or i next pad* or android or itune* or "google play" or ios or wireless* or mhealth or m next health or ehealth or e next health or communication* next technolog* or assistive next technolog* or information next technolog* or ict or "self help" or podcast* or videos or videoconferenc* or videogame* or videophone* or game or games or software* or internet or web or computer* or technolog*):ti
#3 #1 and #2

CINAHL (EBSCO):

S1 (MH "Dementia+")
S2 (MH "Alzheimer's Disease")
S3 (MH "Cognition Disorders+")
S4 TI (alzheimer* OR dementia* OR "cognitive impairment" OR neurocognitive OR "neuro cognitive")
S5 (MM "Mobile Applications")
S6 (MM "Cellular Phone+")
S7 (MM "Computers, Hand-Held+")
S8 (MM "Assistive Technology Devices")
S9 (MM "Telecommunications+")
S10 (MM "Computer Literacy")
S11 (MM "Computer Communication Networks+")
S12 (MM "Software Design")
S13 (MM "Communications Software+")
S14 (MM "User-Computer Interface+")
S15 (MM "Video Games+")
S16 (MM "Web Browsers")
S17 (MM "Therapy, Computer Assisted")
S18 (MM "Computers and Computerization")
S19 (MM "Microcomputers+")
S20 TI (smartphone* OR smart N1 phone* OR "personal digital")
S21 TI (iphone* OR i N1 phone* OR ipad* OR i N1 pad*)
S22 TI ((handheld OR "hand held") N1 (comput* or device*))
S23 TI (android OR itune* OR "google play" OR ios)
S24 TI (mobile OR app OR apps)
S25 TI ((laptop* OR tablet) N2 computer*)
S26 TI (mhealth OR "m health" OR ehealth OR "e health" OR mobile health)
S27 TI ((information* AND communication* AND technolog*) OR ict)
S28 TI ((assistive N1 technolog*) OR (information N1 technolog*))

S29 TI (computer* AND train*)
S30 (s1 OR s2 OR s3 OR s4) AND (s5 OR s6 OR s7 OR s8 OR s9 OR s10 OR s11 OR s12 OR s13 OR s14 OR s15 OR s16 OR s17 OR s18 OR s19 OR s20 OR s21 OR s22 OR s23 OR s24 OR s25 OR s26 OR s27 OR s28 OR S29) Limiter - English Language

PsycINFO (EBSCO):

S1 MM "Dementia" OR MM "AIDS Dementia Complex" OR MM "Dementia with Lewy Bodies" OR MM "Presenile Dementia" OR MM "Semantic Dementia" OR MM "Senile Dementia" OR MM "Vascular Dementia"
S2 MM "Alzheimer's Disease"
S3 TI (alzheimer* OR dementia* OR "cognitive impairment" OR neurocognitive OR "neuro cognitive")
S4 MM "Mobile Devices" OR MM "Cellular Phones"
S5 MM "Computer Literacy"
S6 MM "Electronic Communication" OR MM "Blog" OR MM "Computer Mediated Communication" OR MM "Electronic Learning" OR MM "Social Media" OR MM "Text Messaging"
S7 MM "Communication Systems" OR MM "Internet" OR MM "Telephone Systems"
S8 MM "Online Therapy"
S9 MM "Computer Applications" OR MM "Computer Assisted Instruction" OR MM "Computer Assisted Therapy"
S10 MM "Human Computer Interaction" OR MM "Internet Usage"
S11 MM "Computer Games"
S12 MM "Computer Software" OR MM "Decision Support Systems" OR MM "Groupware" OR MM "Word Processing"
S13 MM "Websites" OR MM "Blog"
S14 MM "Computers"
S15 MM "Microcomputers"
S16 MM "Assistive Technology"
S17 TI (smartphone* OR smart N1 phone* OR "personal digital")
S18 TI (iphone* OR i N1 phone* OR ipad* OR i N1 pad*)
S19 TI ((handheld OR "hand held") N1 (comput* or device*))
S20 TI (android OR itune* OR "google play" OR ios)
S21 TI (mobile OR ((laptop* OR tablet) N2 computer*)
S22 TI (mhealth OR "m health" OR ehealth OR "e health" OR mobile health)
S23 TI ((information* AND communication* AND technolog*) OR ict)
S24 TI (computer* AND train*)
S25 (s1 OR s2 OR s3) AND (s4 OR s5 OR s6 OR s7 OR s8 OR s9 OR s10 OR s11 OR s12 OR s13 OR s14 OR s15 OR s16 OR s17 OR s18 OR s19 OR s20 OR s21 OR s22 OR s23 OR s24) Limiters - English

Ei Compendex (Engineering Village):

#1 (((({Mobile computing} WN CV) OR ({Android (operating system)} WN CV) OR ({iOS (operating system)} WN CV) OR ({Laptop computers} WN CV) OR ({Mobile cloud computing} WN CV) OR ({Mobile devices} WN CV) OR ({Wearable computers} WN CV) OR ({Wireless local area networks (WLAN)} WN CV) OR ({Cellular telephones} WN CV) OR ({Mobile phones} WN CV) OR ({Wireless networks} WN CV) OR ({Mobile telecommunication systems} WN CV) OR ({Text messaging} WN CV) OR ({Smartphones} WN CV) OR ({Video conferencing} WN CV) OR ({Visual communication} WN CV) OR ({Video telephone equipment} WN CV) OR ({Online conferencing} WN CV) OR ({Computer programs*} WN CV) OR ({Computer systems} WN CV) OR ({Computers} WN CV) OR ({Internet} WN CV) OR ({Intranets} WN CV) OR ({Local area networks} WN CV) OR

({Wide area networks} WN CV) OR ({Computer applications} WN CV) OR ({Computer games} WN CV) OR ({Personal computing} WN CV) OR ({Computer software} WN CV) OR ({Web browsers} WN CV)))) AND (1969-2016 WN YR)

#2 ((((((alzheimer*) WN TI) OR ((dementia*) WN TI)) OR (({cognitive impairment}) WN TI)) OR (($neurocognitive) WN TI)) OR (({neuro cognitive}) WN TI)) AND (1969-2016 WN YR)

#3 #1 AND #2

Applied Science & Technology Source (EBSCO):

S1 TI (alzheimer* OR dementia* OR "cognitive impairment" OR neurocognitive OR "neuro cognitive")

S2 DE "Mobile apps" OR DE "Tablet computers -- Mobile apps" OR DE "iPhone (Smartphone) -- Mobile apps"

S3 DE "Mobile app development

S4 DE "Cell phones" OR DE "Camera phones" OR DE "Smartphones"

S5 DE "Pocket computers" OR DE "Handspring Visor (Computer)" OR DE "Smartphones" OR DE "Sony Clié (Computer)"

S6 DE "Handheld video game consoles"

S7 DE "Mobile operating systems" OR DE "iOS (Operating system)"

S8 DE "Personal computers" OR DE "BASIC Stamp computers" OR DE "BeagleBone (Computer)" OR DE "BeagleBone Black (Computer)" OR DE "Buses (Computers)" OR DE "Expansion boards (Microcomputers)" OR DE "MCM/70 (Computer)" OR DE "Microcomputer workstations (Computers)" OR DE "Motherboards (Microcomputers)" OR DE "NEC PC-8200 (Computer)" OR DE "Network computers" OR DE "Pen-based computers" OR DE "Portable computers" OR DE "SC/MP Microcomputer" OR DE "Used microcomputers"

S9 DE "Assistive computer technology" OR DE "Computerized self-help devices for people with disabilities"

S10 DE "Teleconferencing" OR DE "Computer conferencing" OR DE "Videoconferencing"

S11 DE "Videoconferencing -- Computer network resources" OR DE "Videoconferencing -- Software"

S12 DE "Computer literacy"

S13 DE "Computer networks" OR DE "Ad hoc networks (Computer networks)" OR DE "Bandwidth allocation (Networks)" OR DE "Botnets (Computer networks)" OR DE "Bridges (Computer networks)" OR DE "Business enterprises -- Computer networks" OR DE "Computer bulletin boards" OR DE "Computer conferencing" OR DE "Computer network protocols" OR DE "DDLCN (Computer system)" OR DE "Electronic villages (Computer networks)" OR DE "End-to-end delay (Computer networks)" OR DE "Enterprise networks (Telecommunications)" OR DE "GE Mark III (Electronic computer system)" OR DE "Hypercube networks (Computer networks)" OR DE "IP networks" OR DE "Inter-computer links" OR DE "Internet of things" OR DE "Internetworking (Telecommunication)" OR DE "Intranets (Computer networks)" OR DE "Local area networks (Computer networks)" OR DE "Metropolitan area networks (Computer networks)" OR DE "Multicasting (Computer networks)" OR DE "National Geographic Society Kids Network (Computer network)" OR DE "Network operating system" OR DE "Packet switching (Data transmission)" OR DE "Private networks" OR DE "Push technology (Computer networks)" OR DE "Remote access networks" OR DE "Routers (Computer networks)" OR DE "Software-defined networking (Computer network technology)" OR DE "Storage area networks (Computer

networks)" OR DE "Value-added networks (Computer networks)" OR DE "Virtual networks" OR DE "Wide area networks (Computer networks)" OR DE "Wireless personal area networks"

S14 DE "Computer software development" OR DE "Agile software development" OR DE "Application software -- Development" OR DE "B method (Computer science)" OR DE "Cross-platform software development" OR DE "Mobile app development" OR DE "Model-driven software architecture" OR DE "POLYP (Computer system)" OR DE "Rapid application development (Computer software development)" OR DE "Revision control (Computer science)" OR DE "Sandboxes (Computer science)" OR DE "Scrum (Computer software development)" OR DE "Software patterns" OR DE "Software product line engineering" OR DE "Software versioning" OR DE "SysML (Computer science)" OR DE "UML (Computer science)" OR DE "Waterfall model (Computer software development)"

S15 DE "User interfaces (Computer systems)" OR DE "Audio user interfaces" OR DE "Brain-computer interfaces" OR DE "Command-line interfaces" OR DE "Graphical user interfaces (Computer systems)" OR DE "Haptic devices" OR DE "Menus (Computers)" OR DE "Multimodal user interfaces (Computer systems)" OR DE "Web-based user interfaces"

S16 DE "Electronic games" OR DE "Computer games" OR DE "Electronic management games" OR DE "Electronic platform games" OR DE "In-game advertising (Electronic games)" OR DE "Multiplayer games" OR DE "Video games"

S17 DE "Web browsing" OR DE "Web co-browsing"

S18 DE "Computer software" OR DE "ANOHMI (Computer software)" OR DE "Acquisition of computer software" OR DE "Application software" OR DE "Artificial intelligence" OR DE "Business software" OR DE "CLEMMA (Computer system)" OR DE "Children's software" OR DE "Communications software" OR DE "Compilers (Computer programs)" OR DE "Component software" OR DE "Computer assisted instruction -- Authoring programs" OR DE "Computer diagnostic software" OR DE "Computer firmware" OR DE "Computer games" OR DE "Computer poetry -- Software" OR DE "Computer security software" OR DE "Computer software development" OR DE "Computer software installation" OR DE "Computer software metering" OR DE "Computer software usability" OR DE "Computer worms" OR DE "Copyright software" OR DE "Coroutines (Computer programs)" OR DE "Courseware" OR DE "Custom computer software" OR DE "Death -- Causes -- Classification -- Software" OR DE "Decompilers (Computer programs)" OR DE "Device drivers (Computer programs)" OR DE "Disassemblers (Computer programs)" OR DE "Easter eggs (Computer programs)" OR DE "Electronic data processing documentation" OR DE "Electronic spreadsheets" OR DE "Electronic wallets" OR DE "Email software" OR DE "Emulators (Computer programs)" OR DE "Engineering design -- Software" OR DE "Filtering software" OR DE "Freeware (Computer software)" OR DE "Generators (Computer programs)" OR DE "Grammar checkers (Computer software)" OR DE "Groupware (Computer software)" OR DE "Information storage & retrieval systems software" OR DE "Informix software" OR DE "Install programs (Computer programs)" OR DE "Integrated software" OR DE "Intelligent agents (Computer software)" OR DE "Interactive multimedia" OR DE "Internet software" OR DE "Interpreters (Computer programs)" OR DE "Linkers (Computer programs)" OR DE "Loaders (Computer programs)" OR DE "Malware (Computer software)" OR DE "Management -- Software" OR DE "Master graphics software" OR DE "Microcomputer-mainframe links -- Software" OR DE "Middleware" OR DE "Military art & science -- Software" OR DE "Open architecture (Computer science)" OR DE "Open source software" OR DE "Operating systems (Computers)" OR DE "Parallel programs (Computer programs)" OR DE

"Payrolls -- Software" OR DE "Photography -- Software" OR DE "Portable document software" OR DE "Product management software" OR DE "Programming software" OR DE "Reading -- Software" OR DE "Search engines" OR DE "Self-adaptive software" OR DE "Simulation software" OR DE "Social sciences -- Software" OR DE "Software as a service" OR DE "Software shells" OR DE "Source code (Computer science)" OR DE "Spell checkers (Computer programs)" OR DE "Spyware (Computer software)" OR DE "Subroutines (Computer programs)" OR DE "Systems software" OR DE "Technology -- Software" OR DE "Teleprocessing monitors (Computer programs)" OR DE "Text editors (Computer programs)" OR DE "Text-to-speech software" OR DE "Threads (Computer programs)" OR DE "Time management -- Software" OR DE "Translators (Computer programs)" OR DE "Utilities (Computer programs)" OR DE "Word processing software" OR DE "Workflow software" OR DE "microTSP (Computer software)"

S19	DE "Wireless technology in the home" OR DE "Roomba vacuum cleaner"
S20	DE "Wireless communication systems" OR DE "Ad hoc networks (Computer networks)" OR DE "Bit-interleaved coded modulation" OR DE "Bluetooth technology" OR DE "Cognitive radio (Wireless communication systems)" OR DE "FiWi access networks" OR DE "MIMO systems" OR DE "Multipoint distribution service" OR DE "Multiuser detection (Telecommunication)" OR DE "Personal communication service systems" OR DE "Radiotelephone" OR DE "Roaming (Telecommunication)" OR DE "Satellite radio services" OR DE "Software radio" OR DE "Ultra-wideband communication" OR DE "Wireless Application Protocol (Computer network protocol)" OR DE "Wireless Internet" OR DE "Wireless LANs" OR DE "Wireless channels" OR DE "Wireless mesh networks" OR DE "Wireless personal area networks" OR DE "Wireless sensor networks"
S21	DE "Social media" OR DE "Backchannels (Social media)" OR DE "Blogs" OR DE "Computer bulletin boards" OR DE "Online social networks" OR DE "Social media in education" OR DE "Wikis (Computer science)"
S22	TI (smartphone* OR smart N1 phone* OR "personal digital")
S23	TI (iphone* OR i N1 phone* OR ipad* OR i N1 pad*)
S24	TI ((handheld OR "hand held") N1 (comput* or device*))
S25	TI (android OR itune* OR "google play" OR ios)
S26	TI (mobile OR app OR apps)
S27	TI ((laptop* OR tablet) N2 computer*)
S28	TI (mhealth OR "m health" OR ehealth OR "e health" OR mobile health)
S29	TI ((information* AND communication* AND technolog*) OR ict)
S30	TI ((assistive N1 technolog*) OR (information N1 technolog*))
S31	TI (computer* AND train*)
S32	s1 AND (s2 OR s3 OR s4 OR s5 OR s6 OR s7 OR s8 OR s9 OR s10 OR s11 OR s12 OR s13 OR s14 OR s15 OR s16 OR s17 OR s18 OR s19 OR s20 OR s21 OR s22 OR s23 OR s24 OR s25 OR s26 OR s27 OR s28 OR s29 OR s30 OR s31) AND (LA english)

IEEE Xplore (IEEExplore.ieee.org):

1	"Document Title":Alzheimer OR "Document Title":Alzheimers OR "Document Title":"Alzheimer s" OR "Document Title":dementia* OR "Document Title":"cognitive impairment" OR "Document Title":neurocognitive OR "Document Title":"neuro cognitive" OR "Abstract":Alzheimer OR "Abstract":Alzheimers OR "Abstract":"Alzheimer s" OR "Abstract":dementia* OR "Abstract":"cognitive impairment" OR "Abstract":neurocognitive OR "Abstract":"neuro cognitive"

2	"Document Title":mobile OR "Document Title":app OR "Document Title":apps OR "Document Title":smartphone* OR "Document Title":handheld OR "Document Title":"hand held" OR "Document Title":"personal digital" OR "Document Title":iphone* OR "Document Title":"i phone" OR "Document Title":"i phones" OR "Document Title":ipad* OR "Document Title":"i pad" OR "Document Title":"i pads" OR "Document Title":android OR "Document Title":itune* OR "Abstract":mobile OR "Abstract ":app OR "Abstract ":apps OR "Abstract":smartphone* OR "Abstract":handheld OR "Abstract":"hand held" OR "Abstract":"personal digital" OR "Abstract":iphone* OR "Abstract":"i phone" OR "Abstract":"i phones" OR "Abstract":ipad* OR "Abstract":"i pad" OR "Abstract":"i pads" OR "Abstract":android OR "Abstract":itune*
3	("Document Title":cell AND "Document Title":phone*) OR ("Document Title":cell AND "Document Title":telephone*) OR ("Document Title":cellular AND "Document Title":phone*) OR ("Document Title":cellular AND "Document Title":telephone*) OR ("Document Title":smart AND "Document Title":phone*) OR ("Document Title":smart AND "Document Title":telephone*) OR ("Abstract":cell AND "Abstract":phone*) OR ("Abstract":cell AND "Abstract":telephone*) OR ("Abstract":cellular AND "Abstract":phone*) OR ("Abstract":cellular AND "Abstract":telephone*) OR ("Abstract":smart AND "Abstract":phone*) OR ("Abstract":smart AND "Abstract":telephone*)
4	"Document Title":"google play" OR "Document Title":ios OR "Document Title":wireless* OR "Document Title":mhealth OR "Document Title":"m health" OR "Document Title":ehealth OR "Document Title":"e health" OR "Document Title":ict OR "Document Title":"self help" OR "Document Title":podcast* OR "Document Title":videos OR "Document Title":videoconferenc* OR "Document Title":videogame* OR "Document Title":videophone* OR "Document Title":game OR "Document Title":games OR "Document Title":software* OR "Document Title":internet OR "Document Title":web OR "Document Title":computer* OR "Document Title":technolog* OR "Abstract":"google play" OR "Abstract":ios OR "Abstract":wireless* OR "Abstract":mhealth OR "Abstract":"m health" OR "Abstract":ehealth OR "Abstract":"e health" OR "Abstract":ict OR "Abstract":"self help" OR "Abstract":podcast* OR "Abstract":videos OR "Abstract":videoconferenc* OR "Abstract":videogame* OR "Abstract":videophone* OR "Abstract":game OR "Abstract":games OR "Abstract":software* OR "Abstract":internet OR "Abstract":web
5	1 AND (2 OR 3 OR 4)

Scopus (Scopus.com):

#1	TITLE(alzheimer* OR dementia* OR "cognitive impairment" OR neurocognitive OR "neuro cognitive")
#2	TITLE(smartphone* OR "smart phone*" OR "personal digital")
#3	TITLE(iphone* OR "i phone*" OR ipad* OR "i pad*")
#4	TITLE((handheld OR "hand held") W/1 (comput* OR device*))
#5	TITLE(android OR itune* OR "google play" OR ios)
#6	TITLE((laptop* OR tablet) W/2 computer*)
#7	TITLE(mhealth OR "m health" OR ehealth OR "e health" OR "mobile health")
#8	TITLE((information* AND communication* AND technolog*) OR ict)
#9	TITLE((assistive W/1 technolog*) OR (information W/1 technolog*))
#10	TITLE(computer* AND train*)
#11	TITLE(mobile OR app OR apps)

#12 TITLE-ABS-KEY ("amyloid precursor protein*" OR "app/ps1*")
#13 ((#1 AND (#2 OR #3 OR #4 OR #5 OR #6 OR #7 OR #8 OR #9 OR #10 OR #11)) AND NOT #12) AND (LIMIT-TO (LANGUAGE, "English"))

Web of Science (Thomson Reuters):

1 TI=(alzheimer* OR dementia* OR "cognitive impairment" OR neurocognitive OR "neuro cognitive")
2 TI=(smartphone* OR "smart phone*" OR "personal digital" OR iphone* OR "i phone*" OR ipad* OR "i pad*" OR ((handheld OR "hand held") NEAR/1 (comput* OR device*)) OR android OR itune* OR "google play" OR ios OR ((laptop* OR tablet) NEAR/2 computer*) OR mhealth OR "m health" OR ehealth OR "e health" OR "mobile health" OR (information* AND communication* AND technolog*) OR ict OR (assistive NEAR/1 technolog*) OR (information NEAR/1 technolog*) OR (computer* AND train*) OR mobile OR app OR apps)
3 TS=("amyloid precursor protein*" OR "app/ps1*")
4 ((#1 AND #2) NOT #3) AND LANGUAGE: (English)
